# Phosphorylation of biowaste materials for effective removal of organic dye pollutants from aqueous solution; batch and dynamic investigation

**DOI:** 10.1038/s41598-025-18581-y

**Published:** 2025-10-13

**Authors:** AbdElAziz Ahmed Nayl, Ahmed Salah Doma, Aya Gamal Mostafa, Ahmed Ibrahim Abd-Elhamid, Katarína Mosnáčková, Wael Ahmed Arafa, Ahmed Hamad Alanazi, Ismail Mohaamed Ahmed, Hazim Mohamed Ali, Saad Alrashdi, Hisham Fouad Aly, Stefan Bräse, Magda Aly Akl

**Affiliations:** 1 Department of Chemistry, College of Science, Jouf University, 72341 Sakaka, Al Jouf Saudi Arabia; 2 Polymer Department, Advanced Technology and New Materials Research Institute (ATNMRI), City of Scientific Research and Technological Applications (SRTA-City), New Borg Al-Arab, Alexandria 21934 Egypt; 3 Chemistry Department, Faculty of Science, Mansoura University, Mansoura, 35516 Egypt; 4 Composites and Nanostructured Materials Research Department, Advanced Technology and New Materials Research Institute, City of Scientific Research and Technological Applications (SRTA-City), New Borg Al-Arab, Alexandria, 21934 Egypt; 5 Polymer Institute of the Slovak Academy of Sciences, Dubravska cesta 9, 845 41 Bratislava, Slovakia; 6 Hot Laboratories Center, Egyptian Atomic Energy Authority, Nasr, 13759 Egypt; 7 Institute of Biological and Chemical Systems-Functional Molecular Systems (IBCS-FMS), Kaiserstrasse 12, 76131 Karlsruhe, Germany

**Keywords:** Waste tissue, Phosphorylated waste tissue, Dye, Adsorption, Wastewater, Pollution remediation, Environmental chemistry

## Abstract

In this work, the adsorption processes of methyl green (MG-dye) and neutral red (NR-dye) onto phosphorylated waste tissue (P@WT) composite was studied using both equilibrium batch experiments and dynamic flow conditions. Waste tissue (WT) is considered a zero-value, cellulose-rich material, which helps reduce both the cost of the prepared adsorbent and the overall cost of the adsorption process. WT was collected, cut into small pieces, and phosphorylated using urea and sodium phosphate to prepare a low-cost and easily synthesized P@WT composite adsorbent material suitable for practical applications. The prepared materials (WT and P@WT) were characterized using SEM, FTIR, and TGA and investigated as ecofriendly adsorbents to adsorb MG-dye NR-dye from aqueous solutions. The investigated adsorption processes were carried out as a function of different factors, such as adsorbent dose, initial dye concentrations, pH, contact time, Ionic strength, and temperature. The experimental results revealed that the sorption processes obeyed the Langmuir isothermal and pseudo-second-order models. The experimental results were promising at the optimum conditions, wherein the maximum adsorption capacities for batch system process were 445.5, 493.65 mgg^− 1^ for MG-dye and NR-dye; and were 4.54.5, 478.4 mgg^− 1^ for MG-dye and NR-dye for dynamic system process, respectively. Moreover, the adsorbent present suitable kinetics where the equilibrium quickly (3.0 h. for both dyes). The prepared P@WT composite demonstrated high adsorption efficiency and excellent performance in both batch and dynamic systems. Its reusability and effectiveness in binary adsorption further highlight its promising potential. Therefore, this eco-friendly and effective adsorbent can be utilized in wastewater treatment as a potential novel, eco-friendly, low-cost, and effective.

**Supplementary Information:**

The online version contains supplementary material available at 10.1038/s41598-025-18581-y.

## Introduction

Clean water is considered one of the most essential requirements for all living things’ survival^1^. During recent years and with globally rapid population growth in addition to technological advancements, the using of freshwater has surged. Large volumes of wastewater are discharged into surrounding water bodies, which eventually leads to water scarcity and, therefore, must be transported correctly to the usual habitat^2,3^. The major issues contributing to water pollution include wastes from industrial and other human activities^4^. On the other hand, the quick industrial growth in textiles, painting, paper, leather, food, etc., considerably shared the draining of colored materials (such as dye) into the water body. Huge amounts of polluting dyes discharged into wastewater are reportedly due to various industries such as cosmetics, textiles, paper, food coloring, and pulp industries^5^. Such dyes are undesirable even at low concentrations; they color water bodies, obstruct sunlight, and inhibit photosynthesis in the aquatic plants, which induces ecological imbalance^6^. Synthetic dyes are poisonous, carcinogenic, toxic, and seriously threaten aquatic life and ecosystems^7,8^. Organic dyes could be classified as non-biodegradable, toxic, carcinogenic, allergenic, and mutagenic. Neutral red (NR) dye and methyl green (MG) dye are examples of dangerous dyes for humans and ecosystems^9,10^. Due to their high toxicities, environmental issues, and low biodegradability, purification of wastewater from dyes with a suitable approach is essential to reduce their environmental risks and wastewater treatment^11^. Numerous strategies have been investigated to minimize such toxic pollutants, such as liquid-liquid extraction^12^membrane technology^13^biological treatment^14^reverse osmosis^15^catalytic reduction, ion exchange membranes^16^coagulation^17^filtration^18^. The adsorption technique has gained significant attention, as it stands out as a promising and effective approach to treat wastewater contaminated with different types of pollutants^19^. It is simple and economical, does not require complicated systems, and is eco-friendly. However, some adsorbents such as (PANNFs)/CS/PEI (PCP) aerogel^19^adsorptive nanofibrous membranes^20^Fe_3_O_4_@CS-CGSB^21^bentonite^22^zeolites^23^ are expensive, inappropriated thermal stability, and hazard the environment. Recently, the preparation of waste-based adsorbents has attracted significant attention due to their sustainability, cost-effectiveness, and required for a cyclic economy. Moreover, using biowaste materials as adsorbents requires further modification to enhance their adsorption performance^24^. Nowadays, using renewable sources to develop eco-friendly functional materials with high performance is auspicious to drive technological innovations^25^. Recently, cellulosic materials have gained great consideration as eco-friendly, with exceptional mechanical strengths, biodegradability, low-cost, and renewable feedstock to prepare promising adsorbent materials. Their outstanding characteristics make them desirable for various applications^26^. However, due to the intramolecular and intermolecular hydrogen bonding, cellulosic materials are hard to dissolve in common solvents. So, cellulose can be modified by various methods to produce functional cellulosic-based materials that can be integrated with technical applications^27^. Wastes tissue paper (WT) are considered as one of the biomass materials rich in cellulose content and lignin-rich material with a high density of oxygenated function groups^28^making it suitable for grafting with other functionalizing materials. Therefore, the promising characteristics of such bio-waste-based materials such as reusability, structural robustness, and considerably high adsorption efficiency, helping to develop and fabricate sustainable and effective adsorbents^29^. Herein, waste tissue paper was collected and modified with disodium phosphate under specific conditions to prepare phosphorylated (P@WT) composite as adsorbent material. The novel prepared (P@WT) ocomposite material was characterized and applied effectively to remove methyl green (MG)-dye and neutral red (NR)-dye from aqueous media. Different factors affecting the adsorption efficiencies were investigated. Also, the sorption isotherms, kinetic, regeneration, reusability, and adsorption from the binary system and column were studied.

## Materials and methods

### Materials

Waste weep collected from hand dry basket, urea (Central Drug House, 99%), disodium phosphate (Merck, 98%), HCl (Sigma-Aldrich, Lab grade), NaOH (Sigma-Aldrich, Lab grade), methyl green (Alpha Chemika, Lab grade), neutral red (Alpha Chemika, Lab grade).

### Preparation of WT and P@WT composite adsorbents

Waste tissue (WT) was collected from the local markets and cut into small pieces. The cut weep: urea: disodium phosphate: water was mixed with a ratio of (5:18, 5:12, and 2:50) g, stirred for 2.0 h, and dried at 75 °C for 12 h. The resultant solid was thermally treated at 150 °C for another 1.0 h. The obtained material was collected and washed several times with distilled water to remove unreacted material. Finally, the resulting composite P@WT composite was dried at 60 °C for 24 h and stored for further use.

### Characterization

All characterization tools were reported and discussed in the Supplementary Material file (Sect. 2.3.).

### Batch adsorption

A stock solution of 1.0 g/L of each applied dye (MG-dye and NR-dye) was prepared by dissolution of the dye powder in distilled water. The desired concentrations were obtained by further dilution. The adsorption investigations of each dye (MG-dye and NR-dye) were carried out in a stopper bottle containing 25 mL of the dye solution and a specific dose of P@WT composite sorbent. A thermostat shaker was applied to shake the samples at 150 rpm. Different adsorption factors were investigated, such as sorbent dose (0.001–0.030 g/25 mL), dye concentration (25–200 mg/L), pH (2–8), shaking time (15–300 min), NaCl (0.0-0.5 M) and temperature (30–60 °C). At equilibrium, the dye solution easily separated from the solid, and the concentration of residual molecules was determined by UV spectrophotometer MG-dye (λ_max_ = 654 nm) and NR-dye (λ_max_ = 558 nm). The removal percentage of investigated dyes (R, %) and adsorption capacity (qe, mg/g) were determined to be present in Eqs. (1) and (2), respectively.

1 $$\:\text{\%}\text{R}=\frac{\left({\text{C}}_{\text{o}}-{\text{C}}_{\text{t}}\right)}{{\text{C}}_{\text{o}}}\text{x}\:100$$

Where, C_o_ and C_t_ are the initial concentration and the concentration of dye at time t, respectively

2 $$\:{\text{q}}_{\text{e}}=\frac{\left({\text{C}}_{\text{o}}-{\text{C}}_{\text{e}}\right)\text{V}}{1000\text{w}}$$

Where, V is the volume of dye solution (mL) and w is the mass of adsorbent (g.)

Different adsorption models (Kinetics, Isotherm, and thermodynamic) were calculated according to the linear forms presented in Table 1.

**Table 1:** Linear forms of adsorption models.

Study	Model	Linear form	Plot	Slope and intercept	Eq.
Isotherm	Langmuir	$\:\frac{{C}_{e}}{{q}_{e}}=\left(\frac{1}{{q}_{e}b}\right)+\left(\frac{1}{{q}_{e}}\right){C}_{e}$	C_e_/q_e_ vs. C_e_	Slope = 1/q_e_ Intercept = 1/q_e_ b	3
	Freundlich	$\:{log}{q}_{e}={log}{K}_{f}+\frac{1}{n}{log}{C}_{e}$	log Q_e_ vs. log C_e_	Slope = 1**/**n Intercept = logK_f_	4
Kinetic	Pseudo first order	$\:\text{log}({\text{q}}_{\text{e}\:}-\:{\text{q}}_{\text{t}})=\text{log}{q}_{e}+\frac{{\text{K}}_{\text{a}\text{d}\text{s}}\text{t}}{2.303}$	Log (q_e_- q_t_) vs. t	Slope = K_ads_/ 2.303 Intercept = 1/ K_2_ q_e_	5
	Pseudo second order	$\:\frac{t}{{q}_{t}}=\frac{1}{{K}_{2\:}{q}_{e}^{2}}+\frac{t}{{q}_{o}}$	t/q_t_ vs. t	Slope = 1/ q_e_ Intercept = 1/ K_2_ q_e_	6
Thermodynamic		$\:{\varDelta\:\text{G}}^{\text{o}}=\:-\text{R}\text{T}\:\text{L}\text{n}{\text{K}}_{\text{d}}$			7
		$\:\text{L}\text{n}\:{\text{K}}_{\text{d}}=\:\frac{\varDelta\:{\text{S}}^{\text{o}}}{\text{R}}-\:\frac{\varDelta\:{\text{H}}^{\text{o}}}{\text{R}\text{T}}$	Ln Kd vs. 1/T	Slope = ΔH°/R Intercept = ΔS°/R	8

### Determination of point of zero charge (pHpzc) of P@WT composite sorbent

50 mL of (0.1 M) NaCl solutions was charged in various flasks (250 mL), and the initial pH was adjusted by using (0.1 M) HCl and/or NaOH. Thereafter, 0.2 g of P@W was introduced in each flask and left to shake for 48 h. Ultimately, the solutions’ final pH (pHf) was determined. The pHpzc corresponds to the point where pH 0 in the plot of pHi vs. ΔpH (ΔpH = pH_f_− pH_i_).

### Column study

All data obtained in the dynamic study investigated in this work was represented in the Supplementary Material file for different columns used.

## Results and discussion

### Characterization of fabricated adsorbent

#### SEM

**Fig. 1:**
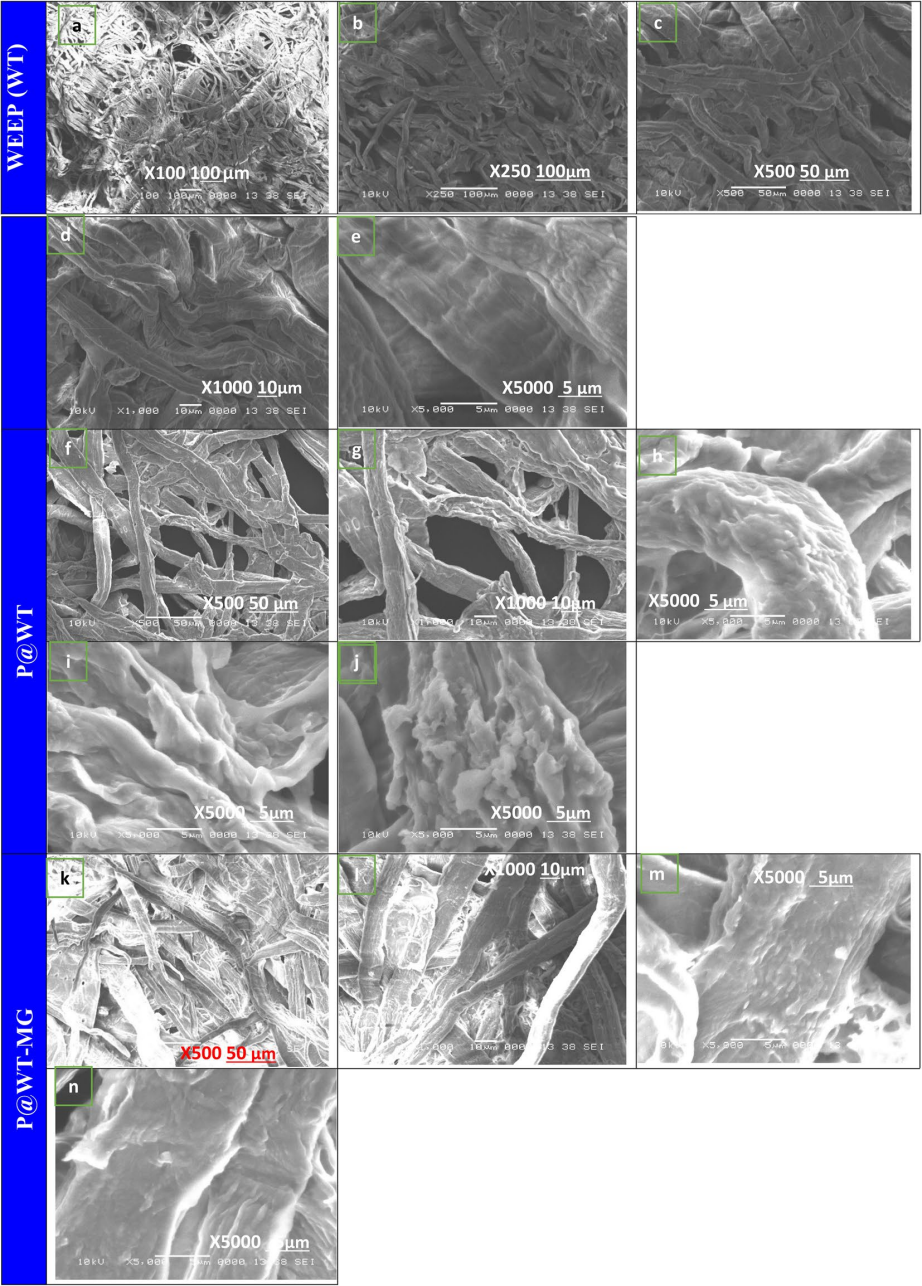
SEM images with different magnifications of (WT), (P@WT), and (P@WT-MG).

The SEM analysis was used to evaluate the morphological structure of WT before and after the impregnation processes. Here, the SEM is used to determine the surface structure of the waste tissue paper (WT), phosphorylated weep (P@WT) composite, and phosphorylated weep after dye adsorption (P@WT-MG), as shown in Fig. 1(a-n). SEM images of WT appear as interconnected fabric structures, as shown in Fig. 1(a-e). Also, it shows a disordered network of cellulosic microfibers with varying diameters and lengths^24^. The waste tissue paper fiber appears as smoothly structured at high magnification as represented in Fig. 1(a-e). The roughness observed on the fiber surfaces can be attributed to impurities such as calcite, lignin, and other fillers added during the production of tissue paper^29^. Following the treatment of waste tissue paper (WT) to synthesize the phosphorylated composite (P@WT), the fiber surface becomes noticeably rougher, as illustrated in Fig. 1(f–j). At high magnification, the substantial presence of P, which may fully coat the samples, leads to a markedly irregular fiber surface accompanied by the formation of surface precipitates^30^. The phosphate groups penetrate the fiber diameters and are homogeneously distributed^30^as illustrated in Fig. 1(f-j). Therefore, the obtained SEM images confirm that the prepared P@WT composite adsorbent is rough, spongy, and porous, thereby most favorable to adsorb the dye species, such as MG-dye species, as illustrated in Fig. 1(l-n). Moreover, they predominantly exhibit quasi-spherical clusters with irregular nanoscale sizes^10^. After MG-dye adsorption, the fiber surface retains its rough texture and illustrates the presence of surface precipitates, as shown in Fig. 1(l-n).

#### FTIR

**Fig. 2:**
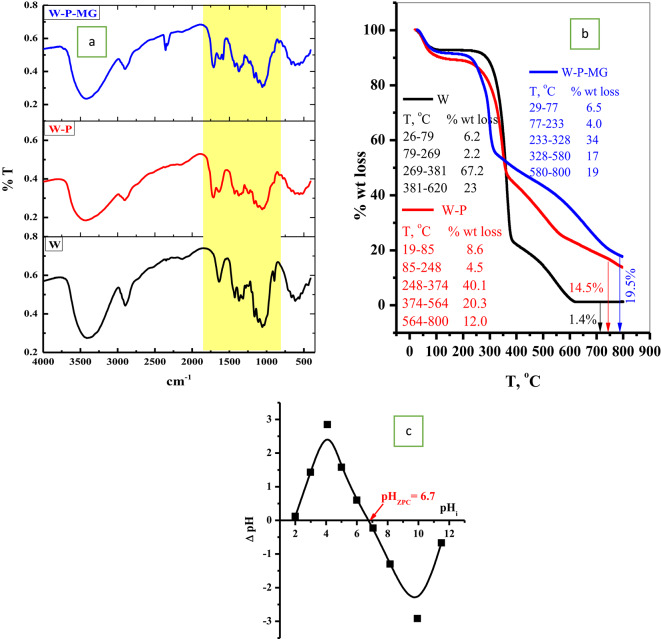
(**a**) FTIR of WT, P@WT, and P@WT -MG., (**b**) TGA of WT, P@WT, and P@WT -MG., and (**c**) point of zero charges of P@WT.

The FTIR is an efficient tool for analyzing the chemical structure of the analyzed materials. The FTIR analysis of the WT, P@WT composite, and P@WT-MG are shown in Fig. 2a. The weep is mainly composed of cellulosic material. Thus, the characteristic peaks of the unmodified WT possess a broad and intense band at 3414 cm^[- [1^ due to water molecule stretching. A medium intense peak appears at 2896 cm^[- [1^ is attributed to the C-H bond stretching of CH_2_ or CH_3_ groups. The peak at 1637 cm^[- [1^ corresponds to water molecule bending^24,29^. The peak of 1415 cm^[- [1^ referred to the C–H deflection angle, and the peaks around 1057 cm^[- [1^ might be due to the vibration of C–O–C and C–O–H stretching of *β*-1,4-glucoside. After modification, the peaks of P@WT have shifted to 3425, 29024, 1614, 1427 and 1058 cm^[- [1^. Moreover, the peak at 1058 cm^[- [1^ becomes broader. On the other hand, a new peak appears at 1714 cm^[- [1^, which may be referred to as the presence of a phosphate groups. Most retained bands indicate that the primary structure of cellulosic materials remained largely intact despite the modifications^24,31^. By introducing the P@W into the aqueous solution of MG-dye, the dye species will interact with the phosphate groups present on the surface of the WT, shifting the peaks to 3416, 2898, 1711, 1618, and 1425 cm^[- [1^.

#### TGA

The thermal stability of the WT, P@WT composite, and P@WT-MG were determined using the TGA tool, as presented in Fig. 2b. The W thermally decomposed through four main stages; the first stage (26–79 °C, 6%) due to losing the physically absorbed water. The second stage (79–269 °C, 2%) referred to the liberation of intermolecular water. The third stage, the main decomposition stage, (269–318 °C, 67%) due to thermal breaking of the glycosyl units^32^. This is followed by another large pyrolysis stage (318–620, 23%) due to the decomposition of the cellulose chain (volatilization of organic substances and hemicellulose decomposition) and incessant slight volatilization^33^. After modification, a new phosphate group will be introduced on the weep fiber. This process will enhance the percent of physically absorbed water (18–85 °C, 8%) and intermolecular water (85–249 °C, 8%). Moreover, the modification step will improve the thermal stability of the P@WT composite in the following stages ((249–374 °C, 40%); (374–563 °C, 20%); (563–800 °C, 12%)), as shown in Fig. 2b. By mixing of P@WT composite with the aqueous solution of MG-dye, the MG species will be adsorbed through the phosphate groups. Therefore, the dye molecules will mask the P@W fiber, which will enhance the thermal stability of the P@WT-MG ((28–77 °C, 6%); (77–232 °C, 4%); (232–326 °C, 34%); (326–580 °C, 17%); (580–800 °C, 19%)), as shown in Fig. 2b.

#### Point of zero charge

The point of zero charge (pH_ZPC_) of the P@WT composite is known as the pH value at which the binding sites of the adsorbent are zero charged; before this point (pH < pHZPC), the binding sites will be positively charged and can interact with anionic species. Beyond this point (pH > pH_ZPC_), the adsorbent binding sites will be ionized and favorable to interact with cationic species^24^. Figure 2c plotted the relationships of ΔpH (pH_fin_ –) vs. initial pH (pH_in_) values. We noted that ΔpH = 0.0 at pHin 6.7 (i.e., pH_ZPC_ = 6.7). In this work, the surface of the P@WT composite will be negative when the pH of the aqueous solution (is ≥ 6.7).

#### EDS

**Fig. 3:**
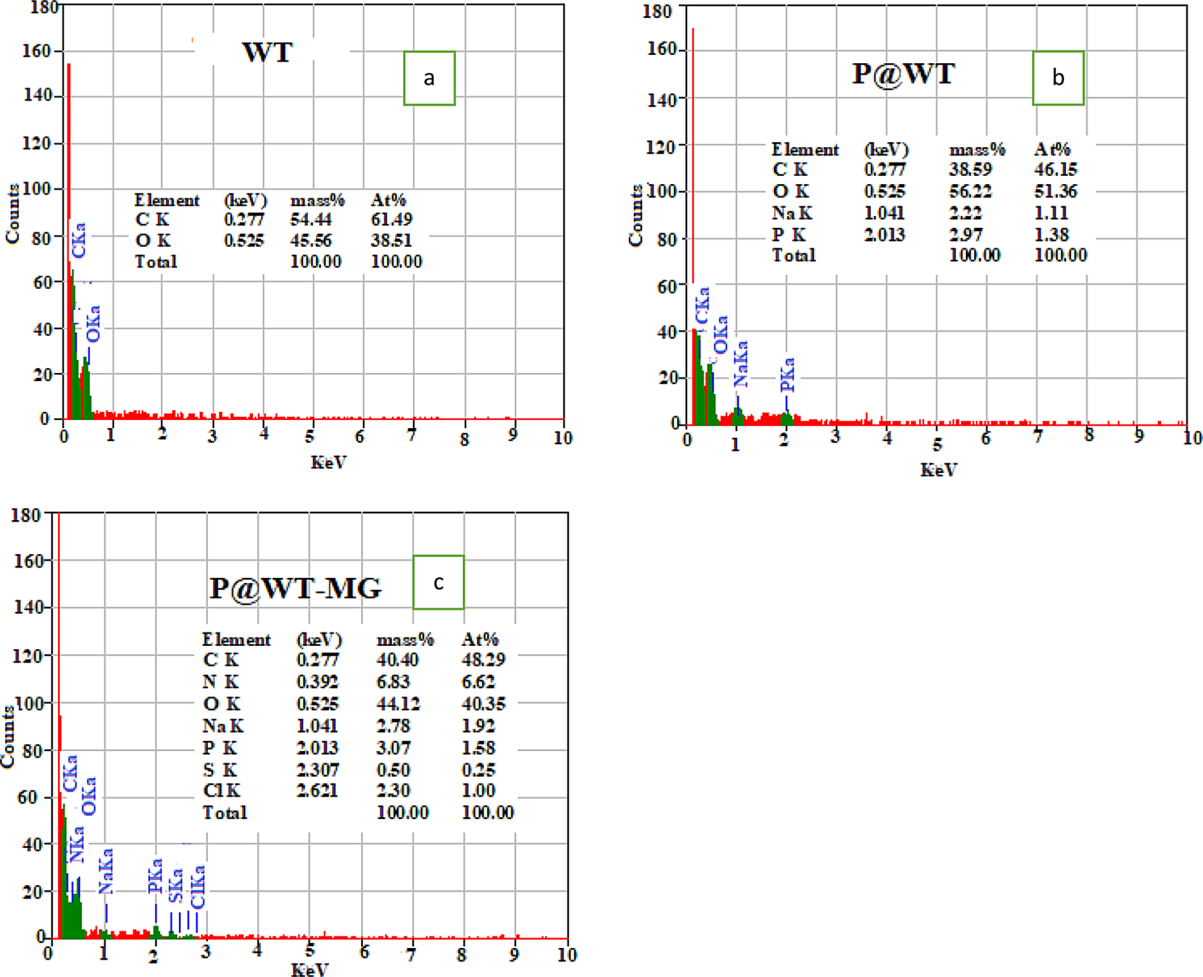
EDS of (**a**) WT, (**b**) P@WT, and (**c**) P@WT -MG.

EDS measurements of WT, P@WT composite, and P@WT-MG were represented in Fig. 3(a-c). The diagram illustrated that the waste tissue (WT) is only consists of C and O, as shown in Fig. 3a, while the fabricated P@WT composite is consists of C, O, Na, and P as illustrated in 3b. This indicate to success the investigated process of fabrication of P@WT composite. After adsorption process of MG-dye onto the prepared P@WT composite, the elemental analysis of P@WT-MG powder was analyzed (as demonstrated in Fig. 3c). The results showed that the elements of N, S, and Cl were observed in the powder of P@WT-MG. This result proves that dye species were adsorbed onto the fabricated P@WT composite under the investigated conditions.

### Adsorption performance by batch experiments

#### Effect of P@WT composite dose

**Fig. 4:**
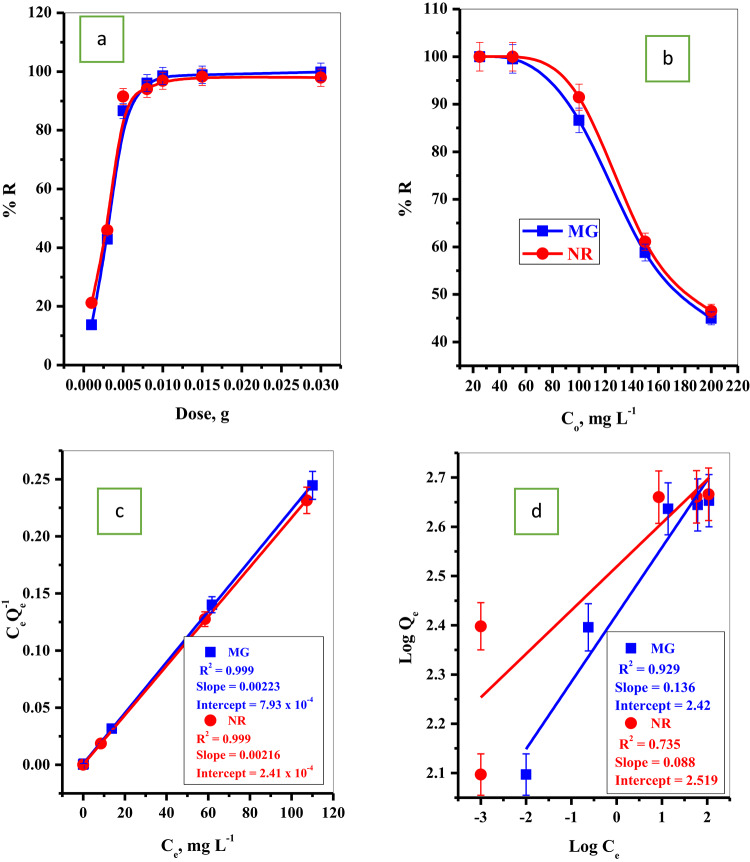
Effect of the (**a**) adsorbent dose/25 mL, (**b**) initial dye (MG and NR) concentration on the adsorption percent of MG and NR dyes, (**c**) Langmuir isotherm plot, and (**d**) Freundlich isotherm plot.

The removal rate of MG-dye and NR-dye species were detected as a function of the variation of the P@WT composite adsorbent amount (0.001–0.03 g/25 mL) as presented in Fig. 4a. As observed, the uptakes percent of the MG-dye and NR-dye dyes improved with increasing the dose of the P@WT composite adsorbent up to the optimum quantity. Beyond this dose, the removal percentage is steady and stable. This could be attributed to the increase in the adsorbent quantity, which will improve the density of the binding sites available to interact with the dye species, leading to an increase in the adsorption percentage. In addition, oxygen-rich segments within the adsorbent structure of P@WT composite can play a vital role in this capability^24^. On the other hand, the concentration of the MG-dye and NR-dye was fixed. Interestingly, despite increasing the P@WT composite dose, no notable improvement in removal efficiency was remarked beyond a certain threshold^24^.

#### Effect of initial MG-dye and NR-dye concentrations

The influence of initial dyes (MG-dye and NR-dye) concentrations on adsorption percent using P@WT composite adsorbent was plotted in Fig. 4b. It is, as the initial concentration of MG-dye and NR-dye increase, the removal percent linearly decreases. This could be explained as at low initial dyes (MG-dye and NR-dye) concentrations, the number of binding sites on the P@WT composite surface was sufficient to interact with the low numbers of the MG-dye and NR-dye species showing high removal percent^34,35^. The number of dye molecules in the aqueous solution will be enhanced with further increases in the initial MG-dye and NR-dye concentrations. In contrast, the number of binding sites is constant. This action will decrease the removal percentage by increasing initial dye (MG-dye and NR-dye) concentrations^34,35^.

The adsorption isotherm describes the pollutant species’ interaction with the active sites on the adsorbent surface. Langmuir (Eq. 3) and Freundlich (Eq. 4) are the most used isotherms to explain the relation between the pollutant concentration and the number of pollutants adsorbed on the adsorbent surface at equilibrium.

Figure 4(c, d) shows the Langmuir and Freundlich adsorption isotherms; the calculated parameters are summarized in Table 2. The correlation coefficient R^2^ = 0.999 for both MG-dye and NR-dye species is larger than the one obtained for Freundlich. This indicates that the adsorption isotherm analysis data was correctly described and in good agreement with Langmuir model with maximum adsorption capacities of 448.43 mg/g for MG dye and 462.96 mg/g for NR dye. These experimental sorption data provided that the sorption of both MG-dye and NB-dye onto the active sites of P@WT composite were homogeneous, uniform, and monolayer processes^6,35^. Furthermore, the applicability of the Langmuir model indicated that the adsorption of both MG dye and NB dye molecules onto the homogeneous sites of the P@WT composite occurred as single molecular layers, (subject-verb agreement) without interactions between adjacent sites or adsorbed molecules^11^. Moreover, the obtained data suggest that the main adsorption sites are predominantly located on the external surface of the P@WT composite. This indicates that external diffusion acts as the rate-limiting step, with the adsorption mechanism proceeding via chemisorption through inner-sphere surface complexation^10^.

#### Effect of pH

**Fig. 5:**
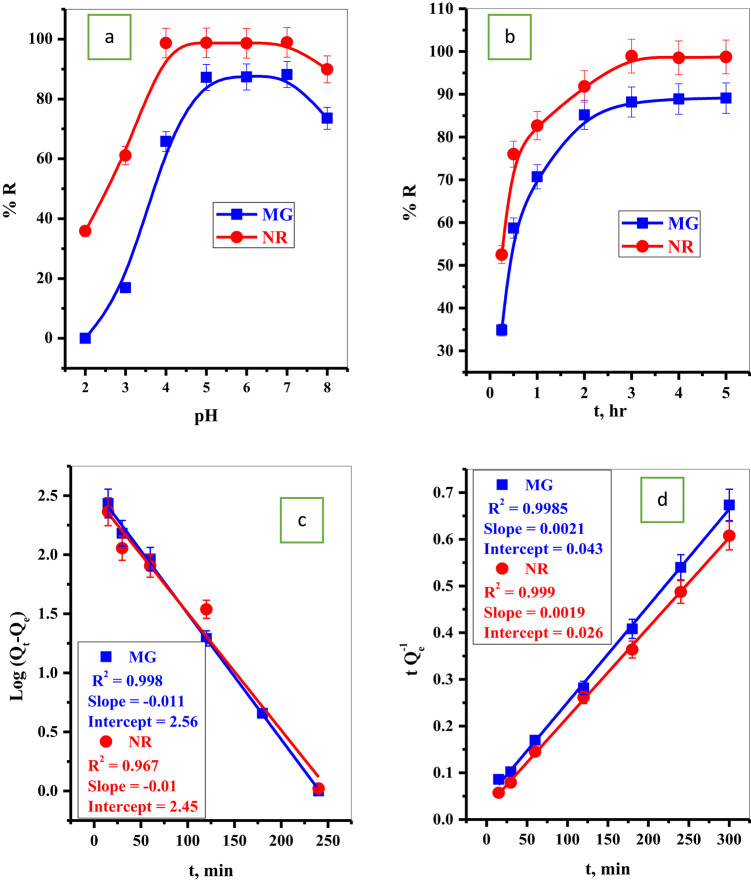
Effect of (**a**) pH value, (**b**) contact time on adsorption percent of MG-dye and NR-dye, (**c**) pseudo first order, and (**d**) second order of MG and NR dye.

The pH of the solution affects the adsorption of pollutants from aqueous solutions. The ionization of the function groups present on the adsorbent surface will affect the pH variation. The plot of the removal percent of the dye species by the P@WT as a function of the pH range (2.0–8.0) is presented in Fig. 5a. The optimum adsorption is shown in the pH range (5.0–7.0). Before this pH range, the adsorption of both MG-dye and NR-dye species decreased^36^. This is because, at low pH values, the concentration of H^+^ ions is high, which will compete with both MG-dye and NR-dye molecules on the adsorption binding sites, decreasing the adsorption percentages^37^. At high pH values, the binding sites become more ionized, creating a favorable environment for adsorption of cationic MG-dye and NR-dye molecules^36^.

#### Effect of contact time

The MG-dye and NR-dye kinetics adsorption curves using P@WT composite sorbent are plotted in Fig. 5b. The plot demonstrated that the equilibrium quickly reached (3.0 h. for both dyes), which may be attributed to the excellent exposure of the active function groups to MG-dye and NR-dye species. The kinetic mechanism was ascribed using the pseudo-first-order (Eq. 5) and pseudo-second-order (Eq. 6). The parameters obtained from fitting pseudo-first order (Fig. 5c) and pseudo-second order (Fig. 4d) are listed in Table 3. The relation coefficient R^2^ of the pseudo-second order for both dyes (MG-dye and NR-dye) is more than the relation coefficient R^2^ related to the pseudo-first order^34^. This result suggested that the adsorption kinetics are excellently described with the pseudo-second-order model, and MG-dye and NR-dye species are chemisorbed over P@WT composite sorbent^34,38^.

#### Effect of ionic strength

**Fig. 6:**
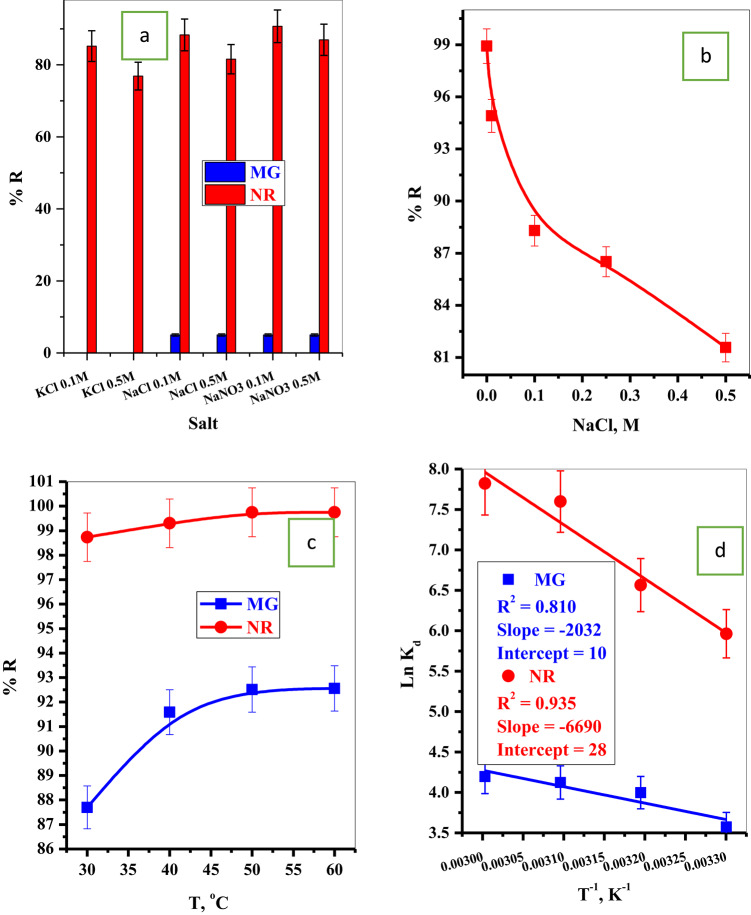
Effect of (**a**) different salts, (**b**) NaCl dose, and (**c**) temperature on the adsorption percentage of MG-dye and NR-dye, and (d)) thermodynamic of MG-dye and NR-dye.

To investigate this parameter, different salts KCl (0.1 & 0.5 M), NaCl (0.1 & 0.5 M), and NaNO_3_ (0.1 & 0.5 M). For NR-dye, the increase in the salt concentration will decrease the removal efficiency, as shown in Fig. 6a. In the case of MG-dye, the addition of KCl to the MG-dye solution will lead to precipitating the MG-dye. On the other hand, the addition of NaCl and NaNO_3_ will highly decrease the removal efficiency to only 5%, as shown in Fig. 6a.

**Table 2:** Parameters of adsorption isotherm models of MG-dye and NR-dye.

Isotherm	Parameters	Dye	
		MG	NR
Langmuir	q_o_ (mg/g)	448.43	462.96
	b (mL/mg)	0.006	0.019
	R^2^	0.999	0.999
Freundlich	K_f_ (mg/g)	263	330.37
	n	7.35	11.36
	R^2^	0.929	0.735

**Table 3:** Parameters of different kinetic models of MG-dye and NR-dye.

Kinetic Model	Parameters	Adsorbing dye	
		MG	NR
	q_e(Exp)_ (mg g^− 1^)	445.5	493.65
Pseudo first order	K_1_ q_e_(cal.) (mg g^− 1^) R^2^	-0.011 0.998	-0.01 0.967
Pseudo second order	K_2_ q_e_(cal.) (mg g^− 1^) R^2^	5.90 × 10^− 5^ 476.19 0.9985	1.386 × 10^− 4^ 526.31 0.999

This can be attributed to the fact that the sorption of MG-dye species onto P@WT occurs by electrostatic interactions through the negatively charged oxygen-containing functionalities cellulosic materials onto P@WT composite and positively charged (= N^+^H-) groups of MG-molecules^37,39^. In the presence of coexisted cations, the adsorption capacities of MG-dye decrease where the presence of these cations at the interfaces of P@WT–water results in the formation of electrical double layers over the surface of the adsorbent due to pairing of charges^37,39^leading to reduce in the adsorption efficiency of the prepared P@WT composite toward MG-dye species. For more explanation, the effect of different concentrations of NaCl (0.0 to 0.5 M) on the removal efficiency of the NR-dye was studied and represented in Fig. 6b. This can be attributed to the presence of coexisted cations, which initiate competitive interactions between these salt ions and NR-dye species at the active sites of P@WT Surfaces and, therefore, exert pronounced influences on the sorption performances^40^.

#### Possible adsorption mechanism

According to the above observations, the mechanisms of the investigated adsorption processes of MG-dye and NR-dye molecules onto P@WT composite can be explained. Both MG-dye and NR-dye are positively charged species and preferably adsorbed on the negatively binding sites. According to the experiment results obtained, (as in section of Point of zero charge), the adsorbent surface will be negatively charged at pH more than 6.7 which will suitable to interact with the positive dyes species. Also, this investigation was confirmed by the studying the effect of the pH of the solution on the adsorption of MG-dye and NR-dye by P@WT composite, in which the increase in the initial pH will enhance the ionization of the active sites (phosphate groups). Consequently, it will induce the negativity of the P@WT adsorbents surface, which can be promoting for excellent interactions with the positively charged dyes species. The adsorption of MG-dye onto the surface of P@WT was confirmed also by using EDS analysis which proved the appearance of N, S, and Cl atoms in the powder of P@WT-MG. Moreover, the interaction of MG-dye with the phosphate groups will cause shift in the phosphate band from 1714 to 1711 cm^− 1^, as illustrated in FTIR analysis. Furthermore, the adsorption of dye species improves the thermal stability of P@WT-MG compared with P@WT composite.

#### Effect of temperature

Figure 6c illustrates the effect of temperature on the removal performance of MG-dye and NR-dye from aqueous solution onto P@WT composite adsorbent. For both MG-dye and NR-dye, an increase in the temperature will follow an increase in the removal percentage. This surface may be due to the increase in the solution temperature, which will increase the dye species motion from the bulk of the solution to the adsorbent surface, enhancing the adsorption efficiency. For the thermodynamic studies, Gibbs free energy change (*ΔG*^*o*^, k J mole^- 1^) is calculated according to Eq. 7. On the other hand, the Enthalpy change (*ΔH*^*o*^, k J mole^- 1^) and Entropy change (*ΔS*^*o*^, J mole^- 1^K ^- 1^) (Eq. 8) were determined from the slope and intercept from plotting 1/T vs. LnK, Fig. 5d. The values of *ΔG*^*o*^, *ΔH*^*o*^ and *ΔS*^*o*^ were listed in Table 4 The negative value of *ΔG*^*o*^ describe a feasible and spontaneous nature of the process. The positive value of *ΔH*^*o*^ presented the endothermic behavior of the adsorption process. The positive values of the *ΔS*^*o*^ are attributed to increasing randomness at the interface of P@WT–water during the adsorption of MG-dye and NR-dye species, which can be attributed to structural modifications on the adsorbent surface occurring throughout the adsorption process^31,37,41^. In addition to the considerable affinities of both MG-dye and NR-dye species towards P@WT composite active sites^37,41^.

**Table 4:** Thermodynamic parameters of the adsorption processes of MG-dye and NR-dye onto P@WT composite.

Dye	T, K	ΔG^o^, k J mole^− 1^	ΔH^o^, k J mole^− 1^	ΔS^o^, J mole^− 1^K^− 1^
MG	303	-9.002	1.70 × 10^4^	38.14
	313	-1.0402		
	323	-1.1072		
	333	-1.1617		
NR	303	-15.021	5.56 × 10^4^	232.79
	313	-17.082		
	323	-20.404		
	333	-21.655		

#### Reusability

**Fig. 7:**
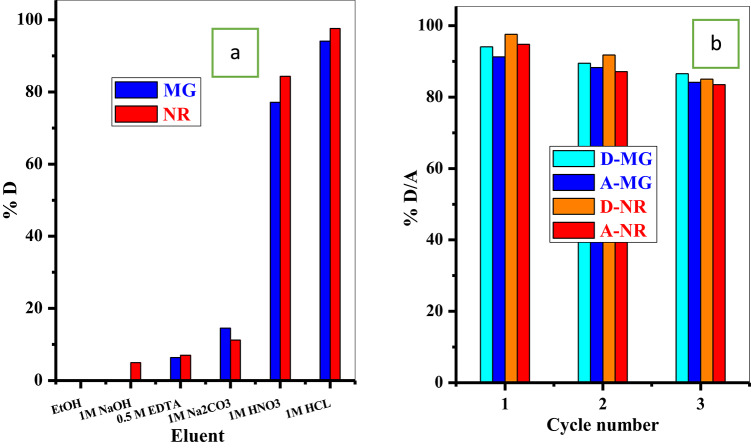
The effect of (**a**) eluent on the desorption percent of MG and NR, (**b**) several cycles on desorption/adsorption percent of MG and NR dyes.

From an economic perspective, the reuse of the adsorbent is of great importance, especially in practical applications. Several eluents were applied to release MG-dye and NR-dye from the P@WT composite adsorbent, as illustrated in Fig. 7a. 1.0 M HNO_3_ and 1.0 M HCl presented both dyes’ best desorption percent (%D). But the 1.0 M HCl possessed the largest %D for both MG-dye and NR-dye; thus, 1.0 M HCl was used for further desorption investigation, as shown in Fig. 7b. Experimental results showed that the desorption percentages for both dyes reduced with the cycle numbers. As a result, the adsorption efficiency was also reduced, as represented in Fig. 7b. This may be due to there being dye species strongly bonded with active sites onto P@WT adsorbent and difficulty releasing during the desorption processes. Therefore, these occupied sites will not be included in the next adsorption process, which will lead to a decrease in the adsorption percentage. Also, the obtained selectivity toward NR-dye species could be due to NR-dye molecular structure, ionic interactions, charge, or stronger affinity to the functional groups on the prepared P@WT composite surface. The experimental results demonstrates that the P@WT adsorbent retains significant efficiency over three cycles, as illustrated in Fig. 7b. In addition to the adsorption/ desorption percentages were slowly decreased with the number of cycles proved the stability of the composite.

#### Adsorption from binary system

**Fig. 8:**
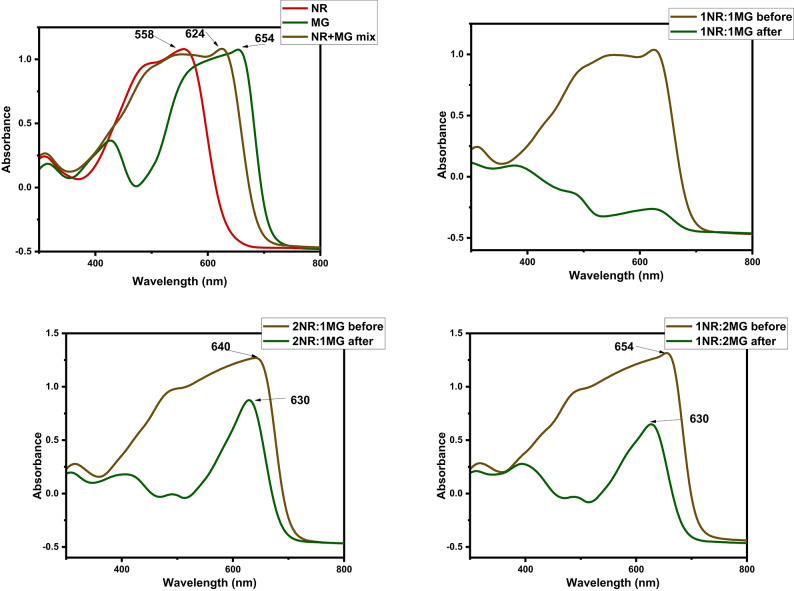
UV-spectrophotometer absorbance of the NR and MG at different concentrations before and after adsorption.

In practice, the dye species present in the mixture of other pollutants. Here, we mixed both the dyes (MG-dye and NR-dye) at different concentration ratios (NR-dye: MG-dye = 50: 50 mg/L, 100: 50 mg/L and 50:100 mg/L), dye solution volume 25 mL and adsorbent dose 0.005 g/25 mL. The absorbance of the binary dye solution was determined before and after adsorption process, as shown in Fig. 8. We noted that at equal concentrations of the two dyes the absorbance highly reduced all over the absorbance range. On the other hand, by using different concentrations of the two dyes (2NR:1MG or 1NR:2MG), we observed an intense band at 630 nm, which may be related to MG dye. Based on this note, we can conclude that the P@WT composite prefers to adsorb NR dye rather than MG dye.

#### Dynamic results

Down-flow configuration; results obtained from dynamic experiments allow us to investigate the applicability of the P@WT composite sorbent. A fixed-bed column was packed with a specific dose of the P@WT composite sorbent and a specific volume of the polluted water (100 ppm) through the top of the column. The resulted clean water was collected, and the concentration of the residue dye was detected, as listed in Table 5.

**Table 5:** The effect of the column type on the adsorption capacities of MG and NR.

Column type	Dose, g	Passed volume (mL)	C_i_ (mg/L)	C_f_ (mg/L)		q_e,_ mg/g	
				MG	NR	MG	NR
1 ml	0.0025	10	100	0	0	400	400
5 ml	0.0025	10	100	0	0	400	400
10 ml	0.0025	10	100	0	0	400	400
25 ml	0.005	25	100	9.1	4.32	454.5	478.4

#### Recent comparison study

Recently, various researches have been published to study and evaluate the adsorption processes of MG-dye and NR-dye from aqueous media onto different types of adsorbent materials. To evaluate and allow validating the performance of our prepared P@WT composite in the adsorption of MG-dye and NR-dye from aqueous media and other adsorbent materials in the literature, their maximum uptake capacities (q_e exp_ (mg g^− 1^) are listed in Table 6^9^. As observed from the cited data, P@WT composite illustrated superior adsorption capacities for adsorption of the investigated cationic dyes. This proves its versatility, promising, feasibility, and potential for different applications and can meet commercial needs for water treatment applications. It is important to note that the adsorption performance data and q_e_(mg/g) values obtained for the various adsorbents materials represented in Table 6 were reported under different experimental conditions, which can influence the results significantly and are intended for general comparison purposes only, not for ranking their performance.

**Table 6:** Comparative evaluation of the maximum adsorption capacities of MG-dye and NR-dye onto P@WT composite and recently reported adsorbent materials.

Adsorbent	Time, min	pH	q_e_, mg/g		Ref.
			MG-dye	NR-dye	
Fe_3_O_4_/sawdust/ 10%MWCNT	30	7	35.5	--	^9^
Nontreated Sejnane Clay Type	10	9	119.6	--	^42^
Purified Sejnane Clay Type	240		149.9	--	
Iron-manganese oxide/GO	160	8	195.7	--	^43^
Chitosan/Fe_2_O_3_/NiFe_2_O_4_	60	8	77.22	--	^44^
MCM-41	60	6	285.70	--	^45^
TiO_2_	45	6.3	384.6	--	^46^
MWCNT/TiO_2_/CS	15	7	269.98	--	^47^
ZIF-76	120	11	96.5	--	^48^
3E-2 H-BCs	60	8	153.6	--	^49^
ECS-MC	120	8	194.4	--	^50^
EC-Cs-OBA-Fe_2_O_3_	30	7.8	47.4	--	^51^
activated residual Dodonaea viscosa (ARDV)	180	6.6	99	--	^52^
MOR zeolite	60	9	40.57	--	^53^
Beta zeolite	60	9	25.05	--	
HY zeolite	60	9	41.54	--	
P@WT	180	4–7	445.5	--	This Work
rGO/SiO_2_ nanocomposites	60	5	--	66.635	^54^
CuO-NP	120	6	--	283	^10^
White clover	100	6	--	113.32	^55^
MDLZ	120	-	--	321.54	^56^
SD	60	6	--	208	^40^
MSD	60	6	--	227	
TAPT-HMIPA-COF	11	7	--	429	^57^
PAsp-g-PAA/Fe_3_O_4_	10	6	--	90.59	^58^
B-C2	240	5	--	145	^59^
*PANI − ZrPB nanocomposite*	240	11	--	56.0	^60^
*MHPMs*	10	7	--	61.5	^61^
P@WT	180	5–7	--	493.65	This Work

The preparation costs of the adsorbent materials including the cost of adsorbent weight unit per each unit of removed adsorbate, cost indices, present value of future cash flows, price of raw resources, are significant aspect to determine suitability of adsorbents to apply to treat wastewaters^62^. The price of raw resources of waste tissue (WT) investigated to prepare phosphorylated P@WT composite is very cheap comparing to various types of raw resources. Table 7 represented a comparative study between cost, recyclability, and efficiency for the prepared P@WT composite and other natural adsorbents materials published recently^45,59,60,63–70^. These published works have investigated that different natural adsorbent materials to remove MG-dye and NR-dye with variation between costs of raw resources, recyclability, and efficiency. The results represented in Table 7 approved that the fabricated P@WT composite adsorbent can be utilized in wastewater treatment as a potential novel, eco-friendly, low-cost, and effective material.

**Table 7:** Comparison between the preparation costs for various types of adsorbents and its adsorption capacities.

Dye	Adsorbent	Cost ($/kg)	Recyclability (No. cycles)	q_e_ (mgg^-1^)	Ref
Neutral red (NR-dye)	Soya waste	≈ 2.0	3	97.12	^63^
	Microcrystalline cellulose from cotton fiber	≈ 5.0	-	83.2	^64^
	Bentonite/carbon composites	≈ 19.0	3	145.00	^59^
	PANI–ZrPB nanocomposite	≈ 30.0	-	54.0	^60^
	Activated carbon chitosan	≈ 42.0	-	36.20	^65^
	Graphene/SiO_2_ Nanocomposites	≈ 32.0	-	66.635	^66^
	Phosphorylated waste tissue (P@WT)	< < 1.0	3	445.5	This work
Methyl green (MG-dye)	Activated residual *Dodonaea Viscosa*	≈ 2.0	-	-	^67^
	Saccharomyces cerevisiae	≈ 16.5	-	20.04	^68^
	Activated carbon prepared from BrachychitonPopulneus fruit shell	≈ 5	-	67.93	^69^
	Functionalised SBA-15	> 10	5	39.4	^70^
	Mesoporous materials MCM-41		4	20.9	^45^
	Phosphorylated waste tissue (P@WT)	< < 1.0	3	493.65	This work

## Conclusion

This work investigated phosphorylation of waste tissue (WT) to prepare phosphorylated (P@WT) composite as a novel adsorbent material. The prepared materials (WT and P@WT) were characterized by multiple techniques, which exhibited the efficiency of the adsorptivity features for the synthesis (P@WT) composite. Additionally, the characterization confirms the cellulosic nature of the prepared composite, highlighting the presence of oxygen functional groups, a combination of irregular and well-defined pores, and predominantly negative surface charges on the adsorbent. The investigated adsorption processes of MG-dye and NR-dye onto the prepared P@WT composite revealed promising processes to remove various organic pollutants from wastewater. In the batch adsorption technique, the fabricated adsorbent materials illustrate promising adsorption performance at optimum conditions (t = 180 min, Dose = 10 mg, V = 25 ml, pH = 4–7, T = 25 °C) and showed superior adsorption capacities of 445.5 and 493.65 mgg^− 1^ toward MG-dye and NR-dye, for batch system process; and were 4.54.5, 478.4 mgg^− 1^ for MG-dye and NR-dye for dynamic system process, respectively. Also, the investigated adsorption processes obeyed the Langmuir isothermal model and pseudo second order model. On the other hand, the dynamic results revealed the prepared phosphorylated (P@WT) composite has considerable adsorption capacities for organic pollutants. The excellent performance of the fabricated adsorbent in its reusability, adsorption from the binary system, and dynamic results suggests other promising advantages compared with other novel adsorbent materials reported in the recent literature. Therefore, P@WT composites can be considered as a potential novel eco-friendly, low-cost, and effective adsorbent to utilize on a large scale in the treatment of wastewater.

## Supplementary Information

Below is the link to the electronic supplementary material.

Supplementary Material 1

## Data Availability

No datasets were generated or analysed during the current study.
